# Integrating knowledge and action: learnings from an implementation program for food security and food sovereignty with First Nations communities within Canada

**DOI:** 10.1186/s13012-023-01291-2

**Published:** 2023-08-12

**Authors:** Ashleigh Domingo, Jennifer Yessis, Kerry-Ann Charles, Kelly Skinner, Rhona M. Hanning

**Affiliations:** 1 Faculty of Health, School of Public Health Sciences, University of Waterloo, 200 University Avenue West, Waterloo, ON N2L 3G1 Canada; 2 Cambium Indigenous Professional Services, Curve Lake, Canada

**Keywords:** Relational approaches, Guiding principles, Knowledge translation, Collaborative research, Participatory action research, First Nations, Indigenous food security, Indigenous food sovereignty, Food systems

## Abstract

**Background:**

Collaborative approaches to knowledge translation (KT) are important for advancing community-engaged research. However, there is a need for examples of participatory approaches that have effectively supported public health research, program development, and implementation with First Nations communities. To strengthen KT with communities, we proposed a set of guiding principles for participatory planning and action for local food system change. Principles emerged from a cross-community analysis of Learning Circles: Local Healthy Food to School (LC:LHF2S) a participatory program (2015–2019) for Indigenous food system action. The objective was to identify guiding principles for participatory planning and action from key learnings and successes on scaling-up of the Learning Circles (LC) model vertically in Haida Nation, British Columbia (BC), and horizontally in three distinct community contexts: Gitxsan Nation, Hazelton /Upper Skeena, BC; Ministikwan Lake. The application of these principles is discussed in the context of our ongoing partnership with Williams Treaties First Nations to support community planning to enhance food security and sovereignty.

**Methods:**

A cross-community thematic analysis was conducted and guided by an implementation science framework, Foster-Fishman and Watson’s (2012) ABLe Change Framework, to identify key learnings and successes from adapting the LC approach. Information gathered from interviews (*n* = 55) and meeting reports (*n* = 37) was thematically analyzed to inform the development of guiding principles. Community sense-making of findings informed applicability in a new community context embarking on food systems work.

**Results:**

Emergent guiding principles for participatory food system planning and action are described within four main areas: (1) create safe and ethical spaces for dialog by establishing trust and commitment from the ground up, (2) understand the context for change through community engagement, (3) foster relationships to strengthen and sustain impact, and (4) reflect and embrace program flexibility to integrate learnings.

**Conclusions:**

Emergent principles offer guidance to supporting Indigenous community-led research and mobilization of knowledge into action. Principles are intended to support researchers and health system administrators with taking a collaborative approach that fosters relationships and integration of community leadership, knowledge, and action for food system change. Application of principles with implementation frameworks can strengthen KT in Indigenous contexts by incorporating community protocols and perspectives in support of Indigenous self-determined priorities.

Contributions to the literature

- The paper responds to growing interests in relational approaches to implementation at a community level and calls made for Indigenous leadership in forging solutions and decision-making in program planning.
- Research findings advance participatory approaches to engage communities in the process and application within Indigenous contexts. Further, we highlight synergies between participatory research and implementation science, as well as opportunities to enhance multi-stakeholder partnerships.
- Application of emergent principles offers ways to strengthen collaboration and research partnerships with communities. Insights into supporting Indigenous-led efforts in program planning including KT in Indigenous contexts is another contribution of the work.

## Background

Participatory processes that can enable effective knowledge translation (KT) have increasingly been recognized as fundamental to initiatives intended to promote health equity and Indigenous peoples’ self-determination within Canada [1–4]. As KT is an integral part of the planning and implementation of programs and services, the broader research community plays an important role in generating knowledge and informing opportunities for application within practice [5–7]. The overall goal of KT or the “knowledge to action” process is to bridge the research-to-practice gap [5, 8]. KT has been described as a “dynamic and iterative process that includes synthesis, dissemination, exchange, and ethically sound application of knowledge to improve the health of Canadians, provide more effective health services and products, and strengthen the health care system” [9]. Within Indigenous health research contexts within Canada, KT is “Indigenously led sharing of culturally relevant and useful health information and practices to improve Indigenous health status, policy, services, and programs” [[10], p.24–25]. Further, Indigenous perspectives are integrated throughout the process [1, 10].

Implementation science^1^ models, frameworks, and theories have offered support for advancing KT efforts, including opportunities to plan for sustainability and scale-up^2^ of promising practices [7, 11–15]. Though interest in implementation science has grown, examples of its application within Indigenous contexts are limited, and strategies and tools for prioritizing community leadership, preferences, and cultural values within program implementation are still needed [16–23]. The use of relational processes such as Indigenous methods and community-based participatory research (CBPR), however, have proved to be promising in supporting both equitable engagement with all partners in the research process and community-led actions in knowledge generation and dissemination [1, 24–28]. Moreover, attention has been drawn to opportunities for the combined use of CBPR and integrated KT^3^ to advance the co-creation and application of research [8].

While the literature on KT, including ways to engage knowledge users in the process, has advanced [8], there remains growing interest in approaches for strengthening collaboration and partnerships to support community-led actions [1, 2, 16–23, 29]. The Learning Circles: Local Healthy Food to School (LC:LHF2S) research within four diverse First Nation contexts presented the opportunity to learn from community participants, NGO partners, and researchers on “what worked” across the 3 years of program implementation. From their rich input, we offer guiding principles to facilitate participatory planning and action for food systems change in Indigenous contexts.

The LC:LHF2S was a participatory initiative (2015–2019) within four community contexts within Canada: Haida Nation, Haida Gwaii, British Columbia (BC); Gitxsan Nation, Hazelton /Upper Skeena, BC; Ministikwan Lake Cree Nation, Saskatchewan; and Black River First Nation, Manitoba [30–33]. Within Canada, Indigenous communities are disproportionately affected by food insecurity and associated health impacts [34–36]. Such health inequities are directly tied to the ongoing impacts of colonization which has disrupted traditional food systems through reduced access to land resources and physical displacement from traditional territories [34–37]. To support community-led food system actions, the LC:LHF2S initiative utilized a participatory model “Learning Circles” (LC) to enhance local and traditional healthy food access, knowledge, and skills. Partnership with broad system stakeholders, including representatives from academia, governments, and community-based and health system organizations with vested interest in environmental sustainability, social justice, equitable food systems, and secure access to food was embedded in the larger project model.

Facilitated by an appointed member of the community, the LC was used to convene a range of Indigenous and non-Indigenous food system actors and leaders, including Elders, traditional food harvesters (e.g., hunters, fishers, gatherers), farmers, food processors, students, parents, and those who work in public health and education. Through community-led LC workshops, participants were involved in a collaborative process to create a vision for food system change, brainstorm and prioritize community needs, and participate in decision-making processes for project development and implementation. Further, the LC process offered the opportunity to adapt planning and monitor project activities. As such, learnings from the process of implementing the LC approach and how it supported the integration of Indigenous knowledge and perspectives to drive actions are described.

We previously applied an implementation science framework, Foster-Fishman & Watson’s (2012) ABLe Change [38], to evaluate the scale-up of the LC model across four diverse contexts [39]. We now offer a set of guiding principles^4^ based on learnings from LC:LHF2S to support a participatory approach that centers Indigenous knowledges and leadership in program action. The objective of the current paper is to describe guiding principles derived from a thematic analysis of learnings from scaling up the LC in four distinct community contexts. Considerations for how emergent principles foster synergies between CBPR and implementation frameworks are discussed. In addition, we offer reflections on how the principles identified can be applied in scaling-up LC in other communities to plan and mobilize actions to strengthen local food security. This knowledge is discussed in the context of our ongoing partnership with Williams Treaties First Nations (Ontario, Canada) to support their planning and actions to advance food security and sovereignty [37].

Findings are intended to support researchers and those occupying positions in the health system (e.g., funders, decision-makers, and non-governmental organizations) with strengthening partnerships with Indigenous communities and with taking a collaborative approach to integrating knowledge and action in food systems work. This can enable culturally meaningful responses in services, programs, and policies aimed at supporting Indigenous health. While efforts are being undertaken to identify promising practices for Indigenous KT [1], this paper draws attention to how relational approaches can be used to integrate Indigenous leadership, methods, and protocols for knowledge generation and application within Indigenous contexts. Findings support broader calls made for implementation efforts aimed at promoting health equity to be guided by collaborative strategies that can support sustainability, cultural safety, and effective transfer of knowledge into practice [1, 14, 16, 25, 40].

## Methods

### LC:LHF2S research partnerships and governance

The LC:LHF2S initiative was developed to support communities with strengthening capacity to enhance local and traditional healthy food access, knowledge, and skills among youth in the community. The program was initially adapted for First Nations contexts in Haida Gwaii, British Columbia, based on the US Farm to School “Learning Labs” [41] model as supported through Farm to Cafeteria Canada [41–44]. The “Learning Circles,” as it became known, used a participatory approach to bring together diverse stakeholders to plan and implement local and traditional school community food actions. Based on promising results in Haida Gwaii (2014–2015) [42–44], the LC model was scaled-up, over a 3-year period (2016–2019) across four First Nations contexts within Canada: Haida Nation, Haida Gwaii, BC; Gitxsan Nation, Hazelton/Upper Skeena, BC; Ministikwan Lake Cree Nation, SK; and Black River First Nation, Manitoba MB. These communities were joined by shared interests in enhancing local, healthy, and traditional foods and skills for youth; however, the food-related actions taken were specific to each community’s capacity, culture, and social context.

The research, funded by the program, Pathways to Health Equity for Aboriginal Peoples, of the Canadian Institutes of Health Research (CIHR), was co-developed with members of communities known to researchers and partners who expressed interest in participating in the initiative. Members of non-government organizations (NGOs), including Heart and Stroke Foundation, Farm to Cafeteria Canada, Storytellers of Hazleton BC, and the Native Women’s Association of Canada—Partner (with CIHR) in Engagement and Knowledge Exchange (NWAK-PEKE) and researchers from the University of Waterloo were also engaged in co-development of the work. In-person meetings in 2014 and 2015 to plan and co-develop the scope of work not only led to a successful CIHR grant proposal spanning 2016–2019, but also extended research partnerships and strengthened community relationships.

The research, “Refining a Scaling up Strategy for Bringing Local Healthy and Traditional Food to School Through Learning Circles in First Nations Communities,” involved vertical scale-up of the LC model in Haida Gwaii focused on increasing community leadership from the people of Haida Nation to support ongoing work in the community [32]. Horizontal scale-up in three other community contexts focused on adapting the LC process to best meet the needs of each community.

Community advisors, LC facilitators from each community, representatives of partnering NGOs, UW researchers (RH, JY), and the project manager provided guidance on governance and conduct of research activities. This engaged group become formally known as the Project Stakeholder Advisory Council. Three graduate-level students were engaged in the work at different stages of initiative planning and implementation.

Further, in each community, a local LC Council was convened with representatives from community health agencies, government, and local schools. The LC Council was responsible for hiring the LC facilitator and worked alongside the Project Stakeholder Advisory Council to support with approval processes in the community, implementation of project activities, and evaluation. The LC facilitator was a community member (Indigenous to the community (*n* = 1), Indigenous from outside of the community (*n* = 2) or non-Indigenous (*n* = 2)) with strong connections to local food systems and school(s)), that led planning with the community members, workshop facilitation, communications, and evaluation activities. The LC facilitator also led engagements with local governance and decision-makers to support the development of research agreements and application of procedures. For example, in Haida Gwaii, British Columbia a Spirit of Collaboration Agreement (Isda ad dii gii isda (S)- Isdaa 'sgyaan diiga isdii (M)) was established with the Haida Foods Committee to support collaborative leadership and decision-making on use and application of project findings.

In support of ethical codes and principles governing the conduct of research activities with Indigenous communities, the First Nations principles of Ownership, Control, Access and Possession [45] were applied in addition to specific protocols identified by the partnering community. Ethics approval to pursue evaluation activities was also obtained from the University of Waterloo Office of Research Ethics (ORE# 30819).

### Learning circles: a participatory model for food system planning

The LC model is supported by a facilitator, appointed by the community, who plans the meeting and invites participants across the local food system to collaborate and prioritize actions for food system change [30–33]. The makeup of the LC varied across communities and time and included Indigenous participants and, in most communities, also non-Indigenous participants. Key participants of LC meetings included food producers, consumers, Elders, community knowledge holders, and representatives from community-based organizations, public health, and schools.

As the LC model is flexible, each community adapted the model in ways that worked for them. However, in most cases, the LC process took place as a full-day in-person workshop in the community where participants engaged in facilitated discussions to build a shared vision, identify goals, exchange ideas, and make connections between people and programs. Further, the group prioritizes ideas (i.e., dotmocracy and discussion) and makes decisions on new food activities to be carried out (e.g., community gardens, food pantry, workshops to build traditional food knowledge and skills). Subsequent meetings have also involved facilitated small group discussions to identify gaps in activities and reflections on what is working well. The number of attendees for LC meetings ranged from 10–15 participants.

In addition to local LC meetings, LC facilitators, community members, and non-Indigenous stakeholders were brought together at four annual gatherings (2015 in Haida Gwaii, 2016 in Hazelton, 2017 in Ministikwan, and 2018 in Black River) [33]. The LC facilitator of the host community led the planning, including invitations to both Indigenous and non-Indigenous representatives, with guidance provided by the host community and project advisory. The number of participants who attended annual gatherings ranged from 15 to 21. Annual gatherings were an opportunity for partnering communities to build relationships, share learnings, celebrate successes, and exchange resources.

### Implementation framework to guide analysis

An implementation science framework, Foster-Fishman & Watson’s ABLe Change Framework (2012), was used to guide an analysis of learnings from LC within and across the four contexts [38]. We now apply the strategic and conceptual elements of ABLe Change to inform the development of guiding principles that can support a participatory approach to planning and implementation that promotes Indigenous values, perspectives, and priorities for action. Specifically, the model addresses both components for building readiness and capacity for the implementation of community projects.

We selected ABLe Change given its emphasis on a strong relational and flexible approach which is important for community-based participatory research. In addition, the iterative, dynamic components built into ABLe Change, along with the emphasis on local engagement, were recognized to be relevant to work with Indigenous communities which requires relation-based approaches [46, 47].

The implementation science framework guided the analysis of the LC process across the four First Nation contexts with the expectation that key themes and emergent principles identified would support implementation within other Indigenous contexts. While we recognize there are Indigenous-specific frameworks such as the First Nations Mental Wellness Continuum Framework and others based on the medicine wheel, these have been used specifically within Indigenous contexts to evaluate outcomes of health services or indicators of health and wellbeing, but not for implementation planning for food system change [48]. As such, the implementation framework used was fitting for identifying key learnings with respect to preparing for implementation within Indigenous contexts. Emergent principles offer key considerations to strengthen a collaborative process for planning and implementation efforts and respond to calls made for greater Indigenous leadership in research, program design, and evaluation processes [49].

### Data sources

Data were collected and analyzed from a range of sources, including interviews with participants of the LC (*n* = 18) and annual gatherings (*n* = 37), LC reports (*n* = 9), meeting minutes (*n* = 15), and activity tracking reports (*n* = 13). Community members, LC facilitators, partners, and research team members participated in annual interviews using a semi-structured interview guide [33] and were conducted by trained community members. A cross-community gathering took place annually (annual gatherings) in each community (4 total) with project advisory members to build relationships, share project stories, engagement experiences, and evaluation activities. Participants from each of the four Annual Gatherings of approximately 15–21 attendees were purposively sampled (~ 8 per gathering) to provide a breadth of interviews. In all cases, the interviewees included LC facilitators and Indigenous community members and advisors, though some non-Indigenous attendees were interviewed as well [33]. A total of 19 people participated over the course of 37 interviews across the four annual gatherings. Specifically, seven attendees participated in one interview and 12 participated in multiple interviews. Interviewees included representatives from a variety of stakeholders such as two research team members, five LC facilitators (note: 1 community had 2 LC facilitators to support a parental leave), seven community members, and five representatives from partnering NGOs (e.g., Heart and Stroke Foundation, Storytellers’ Foundation), dietitians, and PEKE [33].

Interview questions were focused on the experiences of participants at the annual gatherings. In addition, participants were asked to share their reflections with developing and advancing LC goals, including their experiences with moving project activities forward (e.g., challenges, quick wins, and processes that are working well) within the food system, and developments in the community as a result of the LC. Following each LC, the LC facilitator developed a report describing key takeaways and action items from meetings. The LC facilitator also documented notes from conference calls between project partners and emails, which took place throughout the duration of the LHF2S initiative. Written and/or verbal consent was obtained by participants prior to conducting and recording interviews.

### Thematic analysis

Interview transcripts were coded deductively and thematically analyzed according to the adapted version of the ABLe Change Framework. Elements of the framework such as values and norms and power dynamics, emphasized as important for understanding the context for change, helped inform initial codes. For example, while values and preferences for traditional food and skills were captured in the analysis as important for implementation, participant reflections shared on the process for incorporating community knowledge, interests, and understandings informed further interpretations of the data. Other concepts from the framework such as readiness and capacity for building a supportive environment for implementation were also considered. Utilizing a structured phased approach as outlined by Braun and Clarke (2012), data were thematically analyzed across all communities [50–52]. The themes arising from the coded data were organized inductively according to guiding principles for planning and implementing participatory projects with Indigenous communities. Principles were identified to facilitate a collaborative process to understand the context for change within the community that draws on the strengths, knowledge, and values of community members (Table 1).

**Table 1:** Learnings from scaling up LC as a participatory approach for action planning in four First Nations contexts: principles to support community-based action planning and implementation

Guiding principles		Supporting illustrative quotes
**Principle 1: Create safe and ethical spaces for dialog by establishing trust and commitment from the ground up**	**1.1 Recognize and respect community governance, leadership, and protocols**	“Right to land, right to harvest, protocol, how does that work, like one of the circles I remember asking the question- you know teachers were talking about going out and picking soap berries… so then I posed the question, who do you ask to go? How do [you] get out there? Who do you have to ask and what do you have to put into place to take your class and go do that?” [LC participant 11]
		“I think there were lots of things that did happen, like, you know, the land recognition at the beginning, and engaging an Elder. I think those pieces, you know, were done well. We did learn from that, but I think in the broadest sense, um, and maybe that's kind of an impossible expectation on my part…” [LC participant 17]
		“…the importance of including teachings around protocol if we are going to include traditional foods in a school food program. Food is not just food but also medicine. Chiefs are responsible for managing territories.” [LC facilitator 4]
	**1.2 Establish project advisory structures to guide and champion community-driven actions, leadership, and partnerships**	“So, I think it's just, um, for me personally, really reinforced, the fact that we need to do a really good job, I think, from the very outset of, of projects in really letting the community guide the process, I guess. And be part of the development and I think we think we're doing better at that, but I think we can all do so much better” [LC participant 19]
		**“**communities need to be “on board” perhaps bring together an ethics committee” [Learning circle facilitator 1]
		“I’ve been familiar with the [tribal council] for a number of years now, and you know their goals are, you know definitely community benefit oriented. They have a plethora of individuals employed through the [tribal council] that help out with health, education, finance um, and they’re a very large – a corporation. I think the structure mechanism of the [tribal council] is definitely appreciated.” LC facilitator 3]
		“So that would be like one sort of helpful thing. I think um, where I have sort of learned the most in this work is working with local [community] Elders and traditional um, [community] connections – like the connection with the [community] Foods Committee, or these [Indigenous] leadership pieces.” [LC participant 4]
**Principle 2: Understand the context for change through community engagement**	**2.1 Build a shared understanding of values, priorities, and opportunities**	“Food is medicine, is tied to the land, is tied to every aspect of the relations. So, you can’t measure it in terms of like its’ health unless the whole like system is changing to foster a deep, healthy vibrancy on sovereign land.” [LC facilitator 4]
	**2.2 Work within a community’s social, political, and historical context**	“Acknowledge the history of appropriation of Indigenous lands for agriculture. All lands that agriculture takes place on [traditional] territory lands that were appropriated by the Canadian or BC government and reallocated or sold to agriculturalists. Furthermore, agriculture in the form that um’shu’wa use is a new introduction in [community], and is seen as more of an um’shu’wa practice.” [LC facilitator 4]
		And so, for some of the older generation, the connection to farming is like immediately… traumatizing and brings up these memories of this other time. And so, that is a bit of a barrier there, too because there's like resistance to engage with it because, um, of that history…” [LC participant 13]
	**2.3 Identify and build on community supports**	“the Gitxsan Wellness Model and the relationship of lax yip (of that land, Gitxsan knowledge) and otsin (spirit) to the work of connecting young people to their wellbeing through their relationships with culture, relations, food and land. The Wellness Model encompasses a holistic worldview in which the wilp (mother and relations) and the wilksawitx (father and relations) intersect and overlap with the lax yip and the otisin.” [LC participant 24]
		“Each other, share successes and some challenges. We looked at some barriers that we needed to overcome and share, we had a workshop on traditional foods. And what’s really important to keep in mind.” [LC facilitator 1]
**Principle 3: Foster relationships to strengthen and sustain impact**	**3.1 Make connections between people, programs, and processes**	“so I think, I think what’s worked really well is having some kind of core people involved right from the start” [LC facilitator 5]
		“when we did our community planning, parents and community members suggested we have more traditional foods and more healthy foods. And more local people working to prepare the foods” [LC participant 2]
	**3.2 Integrate Indigenous worldviews, perspectives, and values**	“there’s a fear piece about how to talk about current effects of colonialism without sending non-Indigenous people into this fear reactive, defensive place when it’s maybe something somebody hasn’t talked about. And from other work [community organization] is doing from around the work of internal reflection and dialogue that I feel non-Indigenous people need to do…I really feel like, with reconciliation, there is work that both Indigenous and non-Indigenous people need to do.” [LC participant 11]
		“There is still a necessity to sort of unpack, for non-indigenous people to kind of like explore those different narrative and unpack that in the sort of messiness of relationships that some of which have sort of existed between Indigenous and non-indigenous people here for hundreds of years.” [LC facilitator 4]
		“She’s an Elder within the community who will often come and just keep an eye on things. She used to work in the schools as a teacher for a number of years but I remember her coming in and just kind of sitting, she came in halfway, just kind of sat, watching, and you know someone said oh, “we should ask [Name] about this topic on getting kids to eat healthy” and she just said “a potato is a potato. That doesn’t really matter. Get those kids out of school and on the land. That’s what they need to learn.” [LC facilitator 5]
		“Recognize that self-determination is an important or central goal of reconciliation between First Nations and Canadian government. We need to work together to demonstrate that projects need to be centered around Indigenous ways of knowing and adequately support First Nations health and well-being.” [Annual gathering participant]
**Principle 4: Reflect and embrace program flexibility to integrate learnings**	**4.1 Create space for reflection and mutual learnings**	“Share teachings so we are able to learn together and be aware. –create space where we can stand in our own truth, be who we are, and be safe there. –openness-non-judgmental atmosphere in order to allow us to learn. –we have to show up with our baskets full (what is my own culture, where do I come from, what is my truth? engaging as people from a place of truth). –we are always teachers, students, and learners. –respectful dialogue among nations-there is always more to learn.” [Annual gathering participant 6]
		“I think what I’ve learned through this project, that the main barrier is getting everybody to come together and actually just figure out the details.” [LC participant 25]
		“Just to get their experiences- things that worked well, things that didn’t work well when they were engaging with their students. Just to see how much other people are doing. So there is a little bit of solidarity from that to know that like other people are interested in these things and working to do more education around food in schools. So that was really encouraging. So that useful in itself to just have encouragement.” [LC participant 23]
		“You know, their own community supports and where their community's at in terms of being able to keep the project sustainable and continue that work, you know, once funding process is done” [LC participant 19]
		“So in my view what it needed is a pre-initiative capacity assessment that would chronicle in detail the different levels of capacity or rather the different levels of capacity attainment across different domains of capacity….You’ve got an assessment, you place yourself and there’s some menu of options of this version of the model that would best fit your particular circumstances and then you work through the mechanics of how that would work” [LC participant 18]

Members of the team (AD, RH, JY, KS, KAC) critically reviewed and reflected on emergent themes. Indigenous voices were centered throughout community engagements as facilitated by the LC, and project team members reviewed the analysis and interpretation of data collected to ensure the representation of Indigenous voices. From the emergent principles, we worked with a co-author and collaborator from WTFN to consider how communities entering into the process of food system change [37] might translate these principles based on the learnings from LC:LHF2S implementation to questions to support participatory planning and action (Table 2). Nvivo software version 12 Pro (QSR International) was used for coding and analysis.

**Table 2:** Guiding questions to support community-based action planning and implementation

Guiding principles		Questions to support participatory planning and action
**Principle 1: Create safe and ethical spaces for dialog by establishing trust and commitment from the ground up**	**1.1 Recognize and respect community governance, leadership, and protocols**	How will community members be engaged?
		How will leadership and decision-making processes be engaged?
		Is a formal partnership agreement required?
		Is there a mechanism(s) to facilitate trust and commitment between local organizations and partners involved?
		What principles and guidelines should be followed?
		What mechanism(s) need to be in place to manage conflict?
	**1.2 Establish project advisory structures to guide and champion community-driven actions, leadership, and partnerships**	How will local knowledge, values and preferences be integrated?
		What perspectives, knowledge and skills will be helpful?
**Principle 2: Understand the context for change through community engagement**	**2.1 Build a shared understanding of values, priorities, and opportunities**	What areas can be strengthened within your local food system?
		What challenges need to be addressed?
		What are opportunities for change?
	**2.2 Work within a community’s social, political, and historical context**	Are current programs/models/supports grounded in community values and knowledge? If not, why?
		Are current programs/models/supports helpful and meeting community priorities? If not, why?
	**2.3 Identify and build on community supports**	What supports currently exist that can be built upon?
		What programs/supports are needed?
		What skills and experiences are needed to support change efforts?
**Principle 3: Foster relationships to strengthen and sustain impact**	**3.1 Make connections between people, programs, and processes**	Who needs to be engaged?
		Who are key decision makers? How will they be engaged?
		Who are key actors to support on the ground activities? How will they be engaged?
		Who will be impacted by the change? How will they be engaged?
		Who can support the maintenance of project activities and change efforts?
		How are key actors and those impacted by the change connected?
	**3.2 Integrate Indigenous worldviews, perspectives, and values**	What perspectives and knowledge are needed to support change efforts?
**Principle 4: Reflect and embrace program flexibility to integrate learnings**	**4.1 Create space for reflection and mutual learnings**	How are principles and values guiding partnerships?
		What is working well?
		What can be done differently?
		What areas can be improved?

## Results

**Fig. 1:**
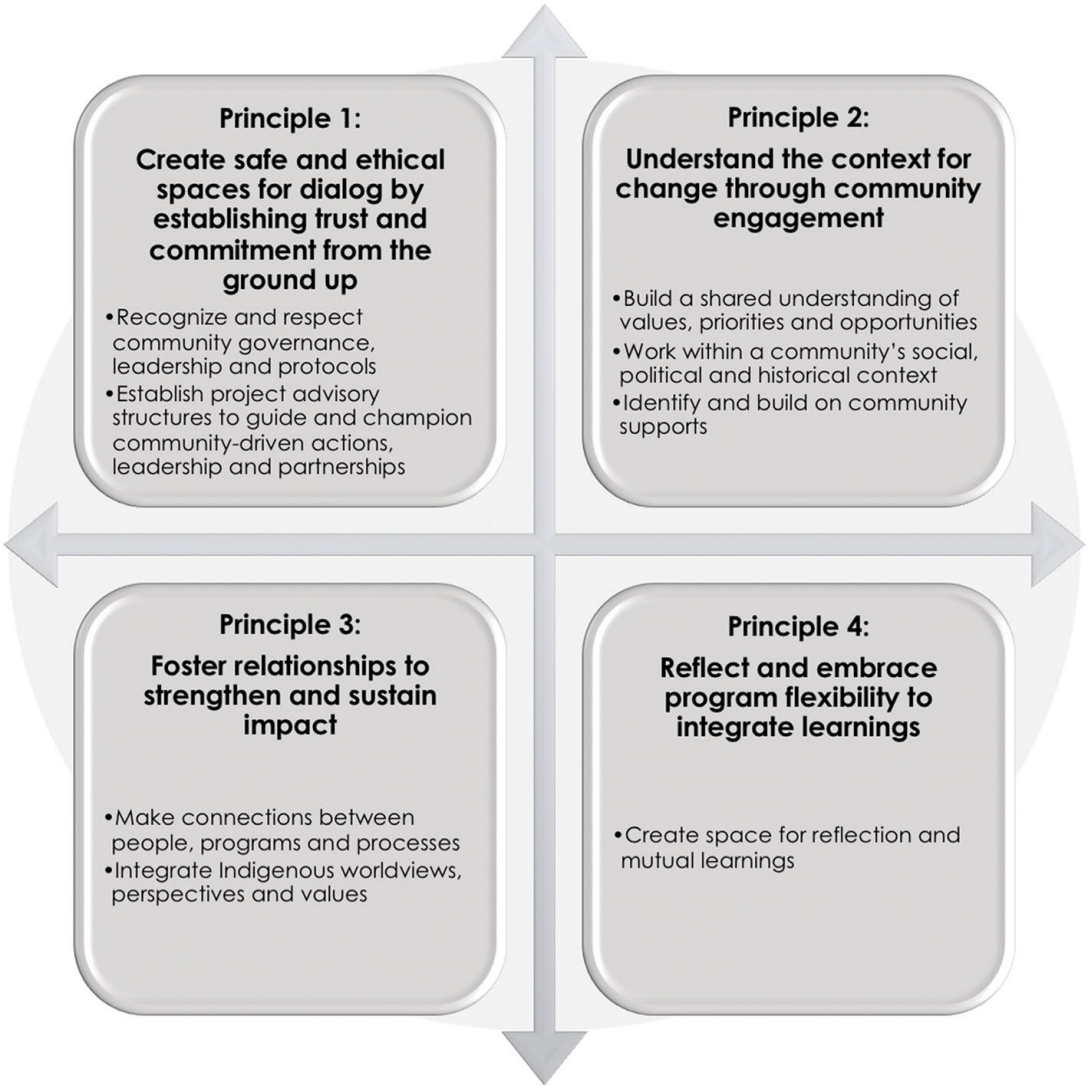
Guiding principles for community-based action planning and implementation

Principles within four main areas emerged from a cross-community analysis of the LC:LHF2S program processes and outcomes. These principles support community-based participatory planning and implementation within First Nations contexts (Fig. 1, Table 1). Each principle is described below and supporting illustrative quotes are presented in Table 1. In addition, Table 2 incorporates a set of questions that can be used to support the application of the principles in participatory planning and action.

### Principle 1: Create safe and ethical spaces for dialog by establishing trust and commitment from the ground up

The process of co-developing an initiative with First Nation community members can be supported by establishing trust and commitment from all partners involved including community members and organizations (Indigenous and non-Indigenous). This was emphasized by participants as an important consideration to creating a safe space for dialog among a broad range of people that bring diverse perspectives and experiences. Key considerations within principle 1 are outlined below as informed by community members.

I think that any project with First Nations you know is contingent, is having relationships with those nations, and those relationships are strengthened through the practical integration of those OCAP principles. Especially when it comes to research projects in particular. [LC participant 19]

#### Recognize and respect community governance, leadership, and protocols

Awareness of community processes for engagement can strengthen approaches for creating safe and ethical spaces for collaboration and dialog in decision-making processes. For example, engaging with a trusted member of the community (e.g., Elder, knowledge holder, community champion) as well as a member of the community’s band or tribal council could help to identify specific guidelines to consider with respect to land use and management practices, engagement with broad members of the community, information sharing and use, and well as other mechanisms to establish a formal partnership with people or organizations outside of the community. Building this initial awareness of governance, leadership, and protocols can foster better ways of working with communities.

[LC facilitator] feels that we are transitioning into a more collaboration with the [First Nation community] and there is a need to develop a better understanding of how we will work together with other partners in this project; protocol rather than agreement. [LC facilitator 1]

#### Establish project advisory structures to guide and champion community-driven actions, leadership, and partnerships

Engaging key people who have deep knowledge of community priorities and who have a strong influence on how decisions are made can support opportunities for grounding the work in community priorities and interests. Identifying who can inform and provide guidance throughout a project can also support fostering trust and commitment from community leadership and drive project activities as informed by the community. For example, each community had established advisory structures to help ensure project scope, objectives, and activities were reflective of community values. Some communities also felt that having a committee involved helped to ensure ethical engagement and integration of community leadership in strategic planning.

### Principle 2: Understand the context for change through community engagement

Working within the LC process to support project planning and implementation, it was clear that enhancing a community’s level of readiness and capacity for change required a deep understanding of community context. Doing so can enable impactful change efforts that are responsive to community priorities, preferences, and Indigenous worldviews.

#### Build a shared understanding of values, priorities, and opportunities

Understanding what changes communities would like to see with respect to their local food systems is a critical step in planning. LC provided a process to facilitate community engagement and brainstorming of current challenges, strengths, and key people to engage in project planning. This process helped to build awareness and understanding of community-identified priorities which enabled communities to see themselves in programs and services. Within the context of LC: LHF2S, this meant convening a range of people to facilitate multi-sector collaboration to identify what gaps exists, what supports and programs are available or are needed, and opportunities for change within the local food system.

So learning how to enjoy foods in a way that’s accessible I think is part of it. How do you make healthy food attractive and delicious and I think that’s a skillset that some, um, many people have lost. And so even in the learning circle there was a cool opportunity to share some of that knowledge back and forth. Where it was like ideas about “oh this is how you can get kids to eat this. [LC participant 23]

#### Work within a community’s social, political, and historical context

Acknowledging the ongoing impacts of colonization and how it has shaped present-day challenges within the community was emphasized. In all communities, people shared the importance of understanding and recognizing the link between colonization and land use practices for food, including farming and loss of land. Indigenous community members identified that racism within off-reserve school communities can be a barrier to the participation of Indigenous people in school-based initiatives to promote food security and food sovereignty. Taking the time to engage deeply with community members to understand and work within the specific context of a community can help to ensure programs developed and delivered are grounded in community values.

It’s about exploring voice and oppression, and how [we] are all sort of, most of our – our ways of being and working in this world is, it’s a – it’s a racialized world, it’s a racialized structure, and that is kind of ingrained and embedded throughout everything. Even here in the [community] where our population is 85 to 90 percent Indigenous. [LC participant 11]

#### Identify and build on community supports

An awareness of community strengths can help to accelerate project planning and action by identifying opportunities to build and expand existing work taking place or where relevant to expand a project to reach more people within the community. For example, one community adopted a wellness model to guide planning efforts and conversations with community members as a way to ensure projects were reflective of community values and perspectives. In addition, understanding what supports exist can help facilitate discussion on what other programs are needed including the resources and people required to inform program development.

There was some work between the [wellness committee] hereditary chiefs. Then the learning circle got involved to help the school apply for some funding and stuff like that….he gave me this model about how this community is approaching food security and how it’s not just based in the school, but based in the school and health, and fisheries and all these other pieces that are going on in their community and how for them it can’t just be based in the school otherwise there is nothing to support it. So that was a really good perspective. [LC facilitator 5]

### Principle 3: Foster relationships to strengthen and sustain impact

Relationships are fundamental to Indigenous ways of knowing and working. Having strong relationships within and outside of the community can help to identify opportunities for partnerships, collective actions and ways to maintain activities to maximize impact as shared by one LC facilitator:

Connecting with other partners and sharing information, and – and just being able to access additional resources, whether they’re financial or otherwise, I feel like we can – we can do that much better collectively. And with strong leadership from [Indigenous community leadership] because then there’s – there’s a great deal more trust. [Learning circle facilitator 1]

#### Make connections between people, programs, and processes

Community engagement through meetings and workshops that bring together a range of people can support opportunities for identifying shared interests and connections with people who have distinct roles within the food system. This can enable communities to identify synergies in work and opportunities to strengthen the coordination of work and services.

It’s taking lots of players and bringing them together. And then they all have their own networks and it’s a really good way to make connections in the food world, or any kind of thing that you’re working on. But it gets people out of their silos and gives an opportunity to work towards common goals. [LC facilitator 1]

#### Integrate Indigenous worldviews, perspectives, and values

Emphasized by all communities was the importance of ensuring project activities, and programs intended to serve the community were grounded within Indigenous worldviews. Where Indigenous and non-Indigenous peoples are engaged in community-level conversations, this can be supported by creating space to center Indigenous voices and perspectives in discussions and decision-making. For example, the LC process was facilitated by a trusted member of the community who would bring people from the community together to plan, share ideas and priorities, and engage in decision-making on food activities. Where Indigenous leadership had a strong presence, the relevance of the LC plans and activities was enhanced.

Making sure First Nations voice is heard. When [non-Indigenous] teachers and principals are part of the LC it may be important to find ways to make sure that voices of community members are heard; perhaps have a co-facilitator who is from the community; also break into smaller groups. In terms of using a talking piece, while there is value in listening to one person speak at a time at some points during the day, there is also a place for dynamic group discussion. [LC facilitator 4]

### Principle 4: Reflect and embrace program flexibility to integrate learnings

#### Create space for reflection and mutual learnings

Learning Circles has been identified as an adaptable strategy across the diverse communities participating in this study [30]. Within each context, opportunities to reflect on achievements and learnings were critical in shaping plans and priorities moving forward and supporting the ongoing relevance of the work. This was recognized as an important consideration to identify successes, challenges, and opportunities to improve project planning and action. This can allow for learnings to be integrated that can strengthen a program and enhance its benefit to communities. Identifying key learnings can inform opportunities to scale up efforts to relevant contexts and where changes may be required to best meet community priorities.

Recognizing the importance of sharing stories of experiences within Indigenous culture and traditions and actively shaping opportunities to reflect and identify learnings can strengthen a program to better meet the needs of those for which it is intended to serve. Utilizing approaches that are iterative and dynamic such as LC can help support this process. LCs are designed so that actions prioritized through a previous LC are discussed and plans can be modified according to ongoing relevance and what worked well (or didn’t).

…in terms of, you know, recognizing, I think things like historical impacts on communities and things like that. Nutrition and what that means and how it can sometimes be a trigger for people in communities. And just expanding our understanding, and growing from that. [LC participant 19]

There’s been a shift from just having farmers in the room, but having harvesters and um, like food knowledge holders, and um, um, the other thing is uh Elders in the room who are sharing information and it’s not just the farmers, you know. That’s a change and then the focus – and when I think back again… in the early years when we were really engaging farmers and farm food getting into the school, that there was also a focus of like farm tours. So students going out to the farm and getting – and looking around and then we kind of worked on getting school greenhouse going, or gardens. Whereas now, there’s a – there’s a real change in like what’s a field trip, or what’s a workshop – like it might be like going seaweed harvesting; or it might be berry picking; or it might be harvesting – like even fishing or hunting trips. [LC participant 4]

## Discussion

In this paper, we propose guiding principles for participatory planning from the context of action for local food system change. These principles are described within four main areas: (1) create safe and ethical spaces for dialog by establishing trust and commitment from the ground up, (2) understand the context for change through community engagement, (3) foster relationships to strengthen and sustain impact, and (4) reflect and embrace program flexibility to integrate learnings. In addition, we outlined questions within each principle to support their application to facilitate participatory planning and action (Table 2). The guiding principles described are based on a cross-community analysis of LC:LHF2S, a program that was scaled up as a participatory approach for actions to strengthen local food systems. The principles are intended to support community-engaged research and implementation with and by Indigenous communities. When applied together, they support a collaborative, iterative, and dynamic process for action planning that welcomes the integration of Indigenous leadership, knowledges, and values.

These principles have their historical roots in action research [53] and community health development [54, 55]. The term “action research” was coined by Lewin [53] who linked community engagement for social planning and action throughout the research process [53]. In addition, the work of Steuart (1969) in the field of community health development has also been recognized to have initiated considerations for the evaluation and integration of research with practice. These two streams of thought have made significant contributions to informing approaches to co-create knowledge with communities and application of research in practice [8, 54].

The commitment to working in partnership with communities is now widely recognized as community-based participatory research (CBPR), which has been used as an umbrella term for such approaches and is employed as a methodology for collaborative and equitable engagement with partners in the research process [8, 56–58]. CBPR has been a long-standing source of guidance on approaches that emphasize collaboration and co-production of knowledge. This focus on collaboration not only aligns with integrated KT practices, but also offers considerations for promoting equitable partnerships and redressing power imbalances, which has the potential for strengthening implementation planning at a community level. Principles for CBPR by Israel, Shulz, Parker, and Becker (1998) emphasize the importance of prioritizing community needs, building on existing strengths, restoring power and control, and reciprocity [58]. Such considerations are supported by the principles brought forth, with opportunities to advance KT within Indigenous contexts through relational approaches that prioritize community interests, leadership, and meaningful research partnerships.

The proposed principles offer guidance to promoting Indigenous community-driven participatory research and mobilization of knowledge to action that draws on strengths offered by implementation science and CBPR. They present opportunities to advance KT through a participatory process between communities and researchers to plan and implement community priorities for action that promote capacity building and equitable partnerships. When used in conjunction with implementation models or frameworks such as ABLe Change and the KTA cycle [5, 38, 59, 60], opportunities for centering Indigenous voices in iterative planning and strengthening collaborative action can be supported. These principles can therefore help fill a gap within implementation frameworks, that when applied within Indigenous contexts, require intentional considerations for community leadership, relationships, preferences, and cultural values in co-planning and implementation. In doing so, this could strengthen implementation within Indigenous contexts by increasing awareness of opportunities for integration of community-specific protocols, knowledge, and preferences throughout the KT process [16–19, 61–64].

When applied within Indigenous contexts, these principles can enable research partners to be guided by community knowledge and welcome opportunities for the use of a variety of techniques and processes as relevant to the communities they are working with. This can help to prioritize community needs and ensure steps are taken to produce and mobilize knowledge into actions that are representative of community values and perspectives. As such, the relational accountability components emphasized in these principles may support broad application within research aimed at promoting health equity within Indigenous contexts.

Within Canada, where food security represents a challenge for many First Nations households in rural and remote communities, protecting traditional and local food systems as a source for healthy food and holistic wellness remains imperative [34–37, 65, 66]. Recognizing the principles outlined in this paper derived from a program specific to advancing food system change, we briefly share reflections on the potential for these principles to be applied in expanding the LC approach to drive actions on food sovereignty and food security. The use of the principles described in this study in new initiatives may help promote Indigenous rights, self-determination, values, and culture. The principles outlined here can be used by those working with communities to support a process that centers community voices and perspectives to drive actions on food security.

For example, in applying principle 1. *Create safe and ethical spaces for dialog by establishing trust and commitment from the ground-up*, in working with a community, one may ask the question “what protocols, principles, and guidelines should be followed?” Opportunities to honor community-specific protocols or integration of Indigenous ways of working and doing to strengthen collaboration, trust, and relationships (e.g., two-eyed seeing, two-row wampum belt, reconciliation pole) [67–69] may therefore be supported. For example, in considering LC as a possible model to inform food system planning with Williams Treaties First Nations (WTFN), alignment with the Seven Grandfather Teachings is important. Accordingly, humility, bravery, honesty, wisdom, truth, respect, and love must guide collaboration and program development [70]. Application of the proposed principles in this context could embrace alignment with the Seven Grandfather Teachings to strengthen meaningful engagement and promote Indigenous ways of working in project planning.

Partnering WTFN communities are currently in the process of utilizing a community-developed tool to inventory strengths and assets (e.g., wild rice beds, fish, market gardens) in the community to support project planning. Findings from the tool will be used by communities to inform ways to improve access to and availability of traditional and local food within their community. As research partners working to support community-based planning, principles outlined in the current study may be applied to facilitate a decolonizing research process for mobilizing change efforts with WTFN communities. Application of guiding principles may also support an exploration of community interests in a range of approaches such as the LC process to accelerate project planning for implementation and food system change. Hence, LC as a model that supports flexible adaptation in advancing access to local healthy and traditional foods, promotes principles of participatory, and decolonizing knowledge to action in program implementation and evaluation. In addition, the co-developed guiding questions (Table 2) may be used to strengthen current processes in place to embed community leadership, build on community strengths, foster strategic linkages between programs and partnerships, and engage communities in decision-making of actions on food security and food sovereignty.

## Conclusion

Based on learnings from the LC:LHF2S program, we offer insights to facilitate participatory planning and action for Indigenous community-based food system change. We propose guiding principles intended to support the integration of knowledge and action in community-based research which draw on strengths from CBPR and implementation science. Application of the proposed principles in conjunction with implementation frameworks such as ABLe Change and KTA can strengthen KT processes by promoting awareness of community protocols and ways to center community perspectives and values. This can enhance program responsiveness to community-identified priorities. Findings are intended to support a range of partners working with Indigenous communities in taking a collaborative approach to center and integrate community knowledge and experiences for local actions on food sovereignty and food security. Such an approach can facilitate responses to provide culturally relevant services, programs, and policies aimed at promoting Indigenous health equity and holistic wellness.

## Data Availability

The data that support the findings of this study are available but restrictions apply to the availability of these data, which were used under license for the current study, and so are not publicly available. Data are however available from the authors upon reasonable request and with permission of partnering communities.

## Footnotes

1 Implementation science emerged as a field to bridge the research-to-practice gap and facilitate the spread of evidence-based practices [7]. Implementation research is the “scientific study of methods to promote the systematic uptake of clinical research findings and other evidence-based practices into routine practice and, hence, to improve the quality and effectiveness of health care” [11, pg.1].

2 Scale-up has been described as efforts to expand the impact of an evaluated intervention. Horizontal scale-up includes replication in other settings; while vertical scale-up includes efforts to support institutionalization [12, 13].

3 Integrated KT has been described as an approach to collaborative knowledge creation, dissemination, and application with the people the research is intended to serve (“knowledge users”) [17].

4 Principles were defined as “a fundamental truth or proposition that serves as the foundation for a system of belief or behavior or for a chain of reasoning” [71].
